# Outcomes of Patients With Atrial Fibrillation Following Thrombectomy for Stroke: A Systematic Review and Meta-analysis

**DOI:** 10.1001/jamanetworkopen.2022.49993

**Published:** 2023-01-06

**Authors:** Hassan Kobeissi, Sherief Ghozy, Trey Seymour, Rishabh Gupta, Cem Bilgin, Ramanathan Kadirvel, Alejandro A. Rabinstein, David F. Kallmes

**Affiliations:** 1 Department of Radiology, Mayo Clinic, Rochester, Minneapolis; 2 University of Minnesota, Twin Cities Medical School, Minneapolis; 3 Department of Neurology, Mayo Clinic, Rochester, Minnesota; 4 Department of Neurologic Surgery, Mayo Clinic, Rochester, Minnesota

## Abstract

**Importance:**

Patients with atrial fibrillation (AF) treated with mechanical thrombectomy (MT) for acute ischemic stroke (AIS) have been reported to experience worse outcomes compared with patients without AF.

**Objective:**

To assess differences between patients with AF and their counterparts without AF treated with MT for AIS, focusing on safety outcomes, clinical outcomes, and baseline characteristics in both groups.

**Data Sources:**

A systematic literature review of the English language literature from inception to July 14, 2022, was conducted using Web of Science, Embase, Scopus, and PubMed databases.

**Study Selection:**

Studies that focused on patients with and without AF treated with MT for AIS were included. Multiple reviewers screened studies to identify studies included in analysis.

**Data Extraction and Synthesis:**

Data were extracted following the Preferred Reporting Items for Systematic Reviews and Meta-analyses reporting guideline to ensure accuracy. Data were pooled using a random-effects model.

**Main Outcomes and Measures:**

The primary outcome of interest was rate of modified Rankin Scale (mRS) scores of 0 to 2 at 90 days. Secondary outcomes of interest included rates of successful reperfusion, defined as thrombolysis in cerebral infarction (TICI) scores of 2b to 3, 90-day mortality, symptomatic intracranial hemorrhage (SICH), and baseline patient characteristics.

**Results:**

Of 1696 initially retrieved studies, 10 studies were included, with 6543 patients. Patients with AF were a mean of 10.17 (95% CI, 8.11-12.23) years older (*P* < .001) and had higher rates of hypertension (OR, 1.89 [95% CI, 1.57-2.27]; *P* < .001) and diabetes (OR, 1.16 [95% CI, 1.02-1.31]; *P* = .02). Overall, there were comparable rates of mRS scores of 0 to 2 between patients with AF and patients without AF (odds ratio [OR], 0.72 [95% CI, 0.47-1.10]; *P* = .13), with significant heterogeneity among the included studies. After sensitivity analysis, the rate of mRS scores of 0 to 2 was significantly lower among patients with AF (OR, 0.65 [95% CI, 0.52-0.81]; *P* < .001). Successful reperfusion rates were similar between the groups (OR, 1.11 [95% CI, 0.78-1.58]; *P* = .57). The rate of SICH was similar between groups (OR, 1.05 [95% CI, 0.84-1.31]; *P* = .68). Mortality was significantly higher in the AF group (OR, 1.47 [95% CI, 1.12-1.92]; *P* = .005).

**Conclusions and Relevance:**

In this systematic review and meta-analysis, patients with AF experienced worse 90-day outcomes, even in the setting of similar rates of successful reperfusion. This was likely associated with greater age and greater rates of comorbidities among patients with AF.

## Key Points

**Question:**

Do outcomes between patients with and without atrial fibrillation (AF) differ following mechanical thrombectomy (MT) for acute ischemic stroke (AIS)?

**Findings:**

This systematic review and meta-analysis including 10 studies with 6543 found that patients with AF had similar rates of modified Rankin Scale scores of 0 to 2, symptomatic intracranial hemorrhage, and thrombolysis in cerebral infarction scores of 2b to 3 compared with patients without AF following MT for AIS; however, patients with AF had significantly greater rates of 90-day mortality. Patients with AF were a mean of 10.17 years older and had a greater number of comorbidities and risk factors for AIS compared with patients without AF.

**Meaning:**

These findings suggest that despite similar rates of successful reperfusion, patients with AF experienced worse outcomes following MT for AIS, which may be attributed to their greater age and greater number of comorbidities.

## Teaser

This systematic review and meta-analysis assesses differences between patients with vs without atrial fibrillation treated with mechanical thrombectomy for acute ischemic stroke, focusing on safety outcomes, clinical outcomes, and baseline characteristics.

## Introduction

Atrial fibrillation (AF) represents a leading comorbidity for large vessel–occlusion acute ischemic stroke (AIS).^1,2^ Mechanical thrombectomy (MT) is the gold standard to treat patients with AIS with large-vessel occlusion, irrespective of cause. Although causality cannot be conclusively determined, previous studies have suggested that outcomes following AIS for patients with AF are worse compared with patients without AF.^3,4^ Additionally, it is estimated that AF is associated with up to a 5-fold increase in stroke risk, and accounts for greater than 15% of all strokes in the United States.^5^ AF rates are known to increase with age, and due to an aging population, there is a projected 2.5-fold increase of AF in the next 40 years.^6^

Therefore, understanding differences in outcomes between patients with AF vs without AF receiving MT is clinically relevant. Although individual studies have compared procedural and functional outcomes between patients with AF vs without AF receiving MT for AIS, to our knowledge, no study has analyzed the existing body of literature to quantify these outcomes between the 2 cohorts.

We conducted a systematic review and meta-analysis of studies that reported outcomes among patients with AF and without AF who underwent MT for AIS to compare procedural outcomes, functional outcomes, and characteristics between these patient populations.

## Methods

### Search Strategy

For this systematic review and meta-analysis, a systematic review of the literature was conducted within Nested Knowledge Autolit software version 1.46 (Nested Knowledge), per the drafted protocol, from inception to July 14, 2022, using PubMed, Embase, Web of Science, and Scopus.^7^ Based on each database, different combinations of possible keywords and/or Medical Subject Headings terms were used for that purpose. Keywords and Medical Subject Headings terms included *stroke*, *cerebral infarction*, *thrombectomy*, *atrial fibrillation*, *AF*, *afib*, *endovascular*, *neurological outcome*, *functional outcome*, *functional independence*, *clinical outcome*, and *therapy*. The full search strategy is provided in the eAppendix in Supplement 1. Moreover, we did an extensive manual search through the references of the included articles to retrieve any missed papers. This study is reported following the Preferred Reporting Items for Systematic Reviews and Meta-analyses (PRISMA) reporting guideline.

### Screening Process

We included all original studies fulfilling our predetermined Population, Exposure, Comparator, and Outcomes approach. The population was patients with AIS with AF, the exposure was mechanical thrombectomy, the control group was patients with AIS without AF, and the primary outcome of interest was modified Rankin Scale (mRS) score of 0 to 2. Secondary outcomes of interest were mortality, thrombolysis in cerebral infarction (TICI) score of 2b to 3, and symptomatic intracerebral hemorrhage (SICH).

We excluded studies in which patients did not have AF, review articles, duplicate studies including the same patients presented in another included study, case reports, case series with fewer than 5 patients, conference abstracts that did not contain the outcomes of interest, and randomized clinical trials. We did not pose any limitations regarding sample size or patients’ characteristics.

Two authors (H.K. and T.S.) performed the title and abstract screening against the predefined criteria. This was followed by a full-text screening of any retained studies of the first screening step. In both stages, the senior author (D.F.K.) was consulted to resolve any conflicts in the decisions.

### Data Extraction

Following a pilot extraction, an extraction sheet was built, and the extraction was performed by at least 2 authors (T.S. and R.G.). The extracted data included study characteristics, baseline data of the included patients, and the aforementioned outcomes of interest. After performing the extraction, a third author (H.K.) did an extensive revision of the extracted data to avoid any prior mistakes.

### Risk of Bias

The Newcastle Ottawa Scale was used to assess the risk of bias, with 2 independent reviewers (H.K. and T.S.) evaluating all studies.^8^ The following rating system was used: good quality: 3 or 4 stars in selection domain, 1 or 2 stars in comparability domain, and 2 or 3 stars in outcome or exposure domain; fair quality: 2 stars in selection domain, 1 or 2 stars in comparability domain, and 2 or 3 stars in outcome or exposure domain; and poor quality: 0 or 1 star in selection domain, 0 stars in comparability domain, or 0 or 1 stars in outcome or exposure domain.

### Statistical Analysis

All analyses were conducted using R software version 4.1.2 (R Project for Statistical Computing) *meta* package version 6.0-0 and *dmetar* package version 0.0.9. We calculated pooled odds ratios (ORs) or mean differences (MDs) and their corresponding 95% CIs using the random-effects model to pool the data in the presence of significant heterogeneity; otherwise, a fixed-effect model was adopted. Heterogeneity was assessed using *Q* statistic and the *I*^2^ test, in which *I*^2^ greater than 50% or *P* < .05 were considered significant.^9^ Whenever there were 10 or more studies, Egger regression test was used to assess the publication bias, with a *P* < .10 considered significant.^10^ In the case of significant heterogeneity, a sensitivity analysis was performed with removal of outlier studies to bring the heterogeneity to an insignificant level. Outlier studies were identified using the method previously described by Viechtbauer and Cheung.^11^ Since the minimum studies per examined covariate in meta-regression is 10 studies,^12^ analogous to the traditional rule of thumb used to minimize the risk of overfitting in regression models,^13^ we could not adjust for different confounders due to the small number of included studies. To compensate, we compared confounders of AF and non-AF groups to determine if there were significant differences.

## Results

### Search and Screening Results

Following the removal of 1251 duplicate records, we retrieved 1694 papers for further screening. We excluded 1678 records through the title and abstract screening stage to retain 16 records for full-text screening. An additional 2 studies were identified via expert recommendation. Finally, 10 studies^14,15,16,17,18,19,20,21,22,23^ with 6543 patients were determined to satisfy our inclusion criteria with the appropriate report of outcomes of interest (eFigure 1 in Supplement 1).

### Study Characteristics and Risk of Bias

Of the 10 included studies, 8 studies^14,16,17,18,19,20,21,22^ used a retrospective design and 5 studies^14,15,19,20,23^ were multicenter studies. The size of the included studies ranged from 78 patients to 4169 patients. Eight studies^14,15,16,17,19,20,21,23^ included in our analysis were determined to have good quality, while 2 studies^18,22^ were determined to have fair quality (eTable in Supplement 1). Patient characteristics, such as age, baseline NIHSS, use of intravenous thrombolytics, and comorbidities, are detailed in the Table.

**Table.** Characteristics of the Included Studies

Source	Design	Setting	Sample size, No.	Baseline NIHSS score		Age, y		Participants, %											
				AF	No AF	AF	No AF	Male		Hypertension		Diabetes		Smoking		CAD		IV TPA	
								AF	No AF	AF	No AF	AF	No AF	AF	No AF	AF	No AF	AF	No AF
Akbik et al,^14^ 2021	Retrospective	Multicenter	4169	16 (6)^a^	15 (7)^a^	76 (11)^a^	65 (15)^a^	42	51	83	70	30	27	NA	NA	NA	NA	46	54
Ždraljević et al,^23^ 2022	Prospective	Multicenter	127	16.7 (5.8)^a^	15.5 (6.2)^a^	74.5 (66.5-79)^b^	61 (50.5-68)^b^	43.5	63.1	88.7	72.3	19.4	15.4	8.1	41.5	NA	NA	17.7	32.3
Huang et al,^15^ 2021	Prospective	Multicenter	245	15 (9-22)^b^	12 (6-18)^b^	74 (67-69)^b^	64 (54-71)^b^	NA	NA	68.3	45.9	NA	NA	16.3	41.8	23.6	13.1	26.8	41
Lasek-Bal et al,^16^ 2022	Retrospective	Single center	417	13 (1-27)^b^	12 (0-43)^b^	75 (68.75-81)^b^	68 (56.5-76)^b^	45	55	NA	NA	NA	NA	NA	NA	NA	NA	71	19
Leker et al,^17^ 2020	Retrospective	Single center	230	18.1 (6.3)^a^	15.8 (6.7)^a^	75 (11.8)^a^	64.5 (15.1)^a^	39.5	56	71.6	65	31.2	32	19.3	30	NA	NA	22	26
Lin et al,^18^ 2020	Retrospective	Single center	83	17.2 (12.1-22.3)^b^	17.9 (11.7-24.1)^b^	72.6 (9.5)^a^	70.9 (17.3)^a^	20	23	65.1	70	25.6	20	18.6	32.5	20.9	20	55.8	37.5
Smaal et al,^19^ 2020	Retrospective	Multicenter	666	17.5 (4.8)^a^	16.5 (5.2)^a^	72.8 (10.1)^a^	63.1 (13.7)^a^	52.2	51	68.8	48.5	21	15.1	NA	NA	NA	NA	73.2	89.2
Fu et al,^20^ 2021	Retrospective	Multicenter	394	18 (12-23)^b^	17 (10-22)^b^	78 (70-83)^b^	67 (37-79)^b^	48.5	58.4	76	67.4	25.7	24.7	11.7	27	NA	NA	31	44.4
Churojana et al,^21^ 2018	Retrospective	Single center	134	17.4 (5.5)^a^	17.1 (6.3)^a^	69.2 (12.9)^a^	60.2 (16.0)^a^	54	60.7	NA	NA	NA	NA	NA	NA	NA	NA	20	22.6
Zhang et al,^22^ 2019	Retrospective	Single center	78	NA	NA	NA	NA	NA	NA	NA	NA	NA	NA	NA	NA	NA	NA	NA	NA

Abbreviations: AF, atrial fibrillation; CAD, coronary artery disease; IV TPA, intravenous tissue plasminogen activator; NA, not applicable; NIHSS, National Institutes of Health Stroke Scale.

^a^ Expressed as mean (SD).

^b^ Expressed as median (IQR).

Use of intravenous thrombolysis was less frequent in the AF group (OR, 0.64 [95% CI, 0.46-0.88]; *P* = .006); however, there was significant heterogeneity among included studies (*I*^2^ = 68%; *P* = .003) (eFigure 2 in Supplement 1). Among the included studies, patients with AF were, an MD of 10.17 (95% CI, 8.11-12.23) years older than their counterparts without AF (*P* < .001); however, there was also significant heterogeneity on this factor among included studies (*I*^2^ = 68%; *P* = .003) (eFigure 3 in Supplement 1). Patients with AF had significantly higher rates of diabetes (OR, 1.16 [95% CI, 1.02-1.31]; *P* = .02), with no significant heterogeneity between studies (eFigure 4 in Supplement 1). Patients with AF had significantly higher rates of hypertension (OR, 1.89 [95% CI, 1.57-2.27]; *P* < .001), with no significant heterogeneity between studies (eFigure 5 in Supplement 1). Patients with AF had similar rates of coronary artery disease (OR, 0.53 [95% CI, 0.14-2.10]; *P* = .37), with no significant heterogeneity between studies (eFigure 6 in Supplement 1). Patients with AF had significantly lower rates of smoking (OR, 0.36 [95% CI, 0.21-0.61]; *P* < .001), with no significant heterogeneity among studies (eFigure 7 in Supplement 1). Patients with AF were less likely to be male than their counterparts without AF (OR, 0.71 [95% CI, 0.60-0.84]; *P* < .001), with no significant heterogeneity among studies (eFigure 8 in Supplement 1).

### Functional Independence 

**Figure 1.**
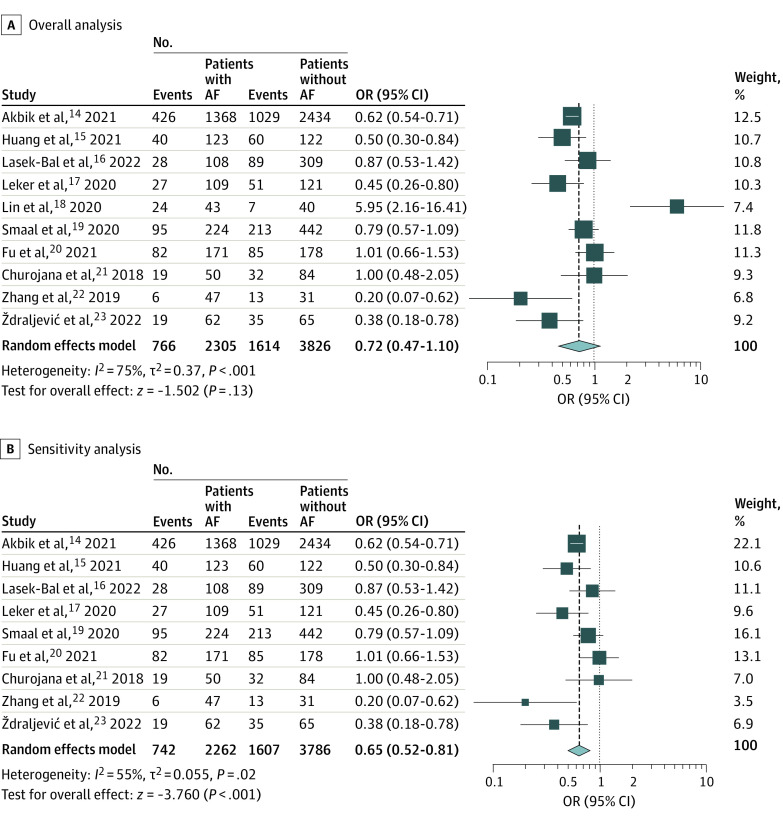
Forest Plot of Odds of Modified Rankin Scale (mRS) Score of 0 to 2 at 90 Days Squares indicate odds ratio (OR), with size of squares indicating weight; horizontal lines, 95% CIs for the ORs; diamond, pooled estimate, with points of the diamond indicating the 95% CI for the pooled estimate. AF indicates atrial fibrillation.

All 10 studies with compared functional independence rates among 6131 patients with AIS with or without AF. There were comparable rates of functional independence (defined as mRS score of 0-2) at 90 days between groups (OR, 0.72 [95% CI, 0.47-1.10]; *P* = .13); however, there was significant heterogeneity among studies (*I*^2^ = 75%; *P* < .001) (Figure 1A). Egger regression test showed no publication bias (eFigure 9 in Supplement 1).

On sensitivity analysis excluding the outlier study (Lin et al^18^) (eFigure 10 in Supplement 1), the heterogeneity of the remaining studies remained significant (*I*^2^ = 55%; *P* = .024). The results of the sensitivity analysis revealed significantly lower rates of functional independence among patients with AF compared with patients without AF (OR, 0.65 [95% CI, 0.52-0.81]; *P* < .001) (Figure 1B).

### Successful Reperfusion

**Figure 2.**
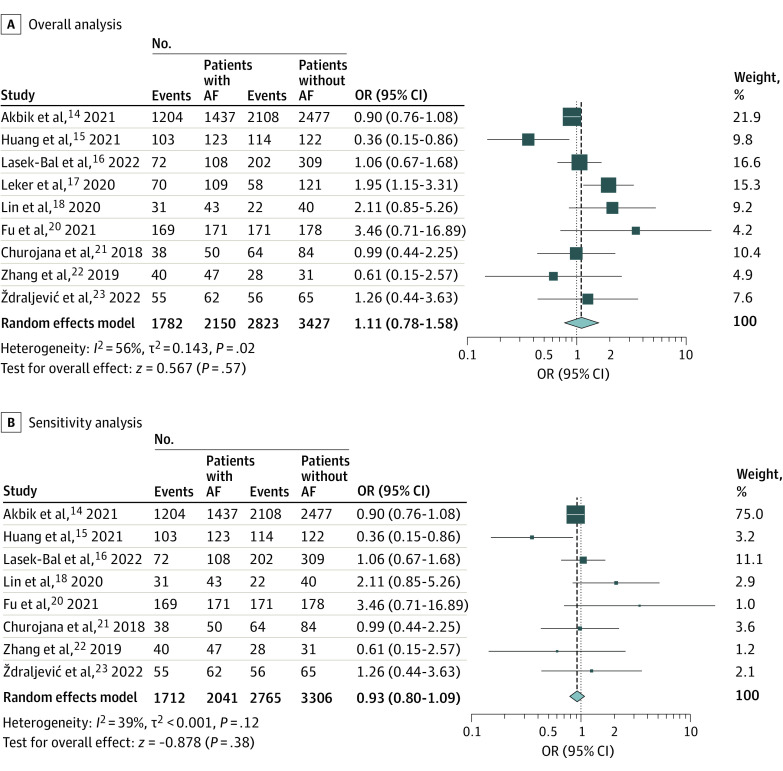
Forest Plot of Odds of Thrombolysis in Cerebral Infarction Score 2b or 3 Squares indicate odds ratio (OR), with size of squares indicating weight; horizontal lines, 95% CIs for the ORs; diamond, pooled estimate, with points of the diamond indicating the 95% CI for the pooled estimate. AF indicates atrial fibrillation.

Successful reperfusion rates (defined as TICI score 2b-3) were reported in 9 studies^14,15,16,17,18,20,21,22,23^ including 5577 AIS patients who underwent MT. Successful reperfusion rates were similar between patients with and without AF (OR, 1.11 [95% CI, 0.78-1.58]; *P* = .57); however, there was significant heterogeneity among studies (*I*^2^ = 56%; *P* = .02) (Figure 2A). On sensitivity analysis, no change in successful reperfusion rates was observed after removal of the outlier study (Leker et al^17^) (eFigure 11 in Supplement 1) (OR, 0.93 [95% CI, 0.80-1.09]; *P* = .38), with no significant heterogeneity among the remaining studies (Figure 2B).

### SICH and Mortality Rates

**Figure 3.**
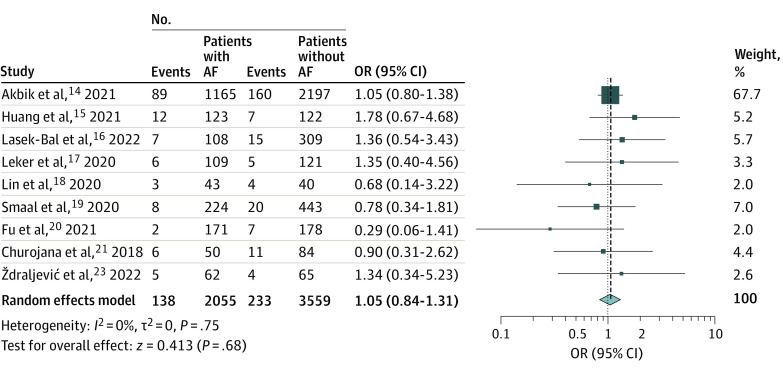
Forest Plot of Odds of Symptomatic Intracranial Hemorrhage Squares indicate odds ratio (OR), with size of squares indicating weight; horizontal lines, 95% CIs for the ORs; diamond, pooled estimate, with points of the diamond indicating the 95% CI for the pooled estimate. AF indicates atrial fibrillation.

Nine studies^14,15,16,17,18,19,20,21,23^ with 5614 patients compared the rates of SICH after MT between patients with and without AF. Rates of SICH were comparable between groups (OR, 1.05 [95% CI, 0.84-1.31]; *P* = .68), with no heterogeneity observed across studies (*I*^2^ = 0%; *P* = .75) (Figure 3).

**Figure 4.**
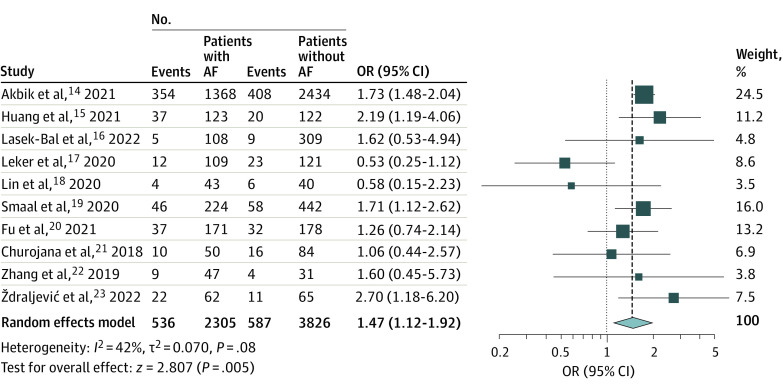
Forest Plot of Odds of Mortality at 90 Days Squares indicate odds ratio (OR), with size of squares indicating weight; horizontal lines, 95% CIs for the ORs; diamond, pooled estimate, with points of the diamond indicating the 95% CI for the pooled estimate. AF indicates atrial fibrillation.

Ten studies^14,15,16,17,18,19,20,21,22,23^ with 6131 patients compared the rates of 90-day mortality between patients with and without AF. Mortality was significantly higher in the AF group (OR, 1.47 [95% CI, 1.12-1.92]; *P* = .005), with no significant heterogeneity observed (*I*^2^ = 42%; *P* = .08) (Figure 4). In addition, there was no publication bias among the included studies (eFigure 12 in Supplement 1).

## Discussion

In this systematic review and meta-analysis, we found that patients with AIS and AF had significantly higher rates of mortality and significantly lower rates of functional independence after MT compared with patients without AF, even in the setting of comparable rates of successful reperfusion. These findings are important because they provide context for the worse outcomes observed in patients with AF compared with patients without AF.

Previous studies have reported that AF is significantly associated with worse 90-day outcomes, higher rates of mortality, and comparable rates of successful reperfusion.^14,15,16,17,18,19,20,21,22,23,24^ Yet, these previous studies were individually much smaller than the cohort included in our pooled analysis. Interestingly, 1 study^18^ included in our analysis could be considered an outlier regarding functional independence rates. Lin et al^18^ reported a significantly better rate of functional independence in patients with AF. This study was a single-center study with a small sample size of 83 patients, limitations that could have influenced the reported results. Again, the study by Zhang et al^22^ had a small sample size of 78 patients, which likely contributed to the difference in outcomes.

The worse functional outcomes and higher rates of mortality observed in patients with AF is interesting because the procedural outcomes were comparable between groups. Thus, the worse outcomes in patients with AF cannot be attributed to technical success rates. There are several possible explanations for the differences in functional outcomes. As a population, patients with AF tend to have higher rates of comorbidities, including hypertension, dyslipidemia, heart failure, diabetes, smoking, asthma, chronic obstructive pulmonary disease, history of myocardial infarction, arrythmia (other than AF), and valvular heart disease.^25,26^ These trends held true in our analysis, with patients with AF exhibiting higher rates of hypertension and diabetes. We hypothesize that the worse outcomes seen in patients with AF was because these patients were typically less healthy than their counterparts without AF.

Patients with AF also tend to be older, and it has been previously shown that younger patients have better outcomes following MT for AIS.^27,28^ All studies in our systematic review and meta-analysis reported that patients with AF were older than their counterparts without AF, which is consistent with patients with AF in general. In fact, 70% of people with AF in Australia, Europe, and the US are aged 65 years or older.^29^ In our study, patients with AF were approximately 10 years older compared with patients without AF. It is possible that patients with AF have worse outcomes because of these traits, instead of AF itself contributing to the worse outcomes.

Patients who have AF tend to present with a lower Alberta Stroke Program Early CT Score (ASPECTS)^30^ and more severe deficits at onset of stroke.^14^ Previous literature has indicated that patients with a high ASPECTS are more likely to achieve favorable outcomes, and that a low ASPECTS is a factor associated with unfavorable outcomes in stroke.^30^ Although we could not perform a formal analysis to account for these differences, these trends were present in all studies included in our meta-analysis. Rates of intravenous thrombolysis were lower among patients with AF treated with MT compared with their counterparts without AF. This is unsurprising, given that patients with AF more often receive anticoagulant drugs, and anticoagulation represents a contraindication for intravenous thrombolysis. Yet, this baseline difference could have contributed to the differences in outcome.^31,32^

### Limitations

This study has limitations. We were unable to perform meta-regression to test for the outcome associated with different individual confounders, such as age and diabetes, although as a group, patients with AF had more comorbidities than their counterparts without AF.^25^ We did not have access to patient-level data, which also limited the analysis we were able to perform. In particular, we were not able to account for severity of stroke at presentation and time from stroke onset to reperfusion.

## Conclusions

In this systematic review and meta-analysis of 10 studies,^14,15,16,17,18,19,20,21,22,23^ patients with stroke from large-vessel occlusion and AF experienced worse 90-day outcomes than patients without AF, despite having similar rates of successful reperfusion. This is possibly associated with older age and more comorbidities among patients with AF.

## Notes

Supplement 1. **eFigure 1.** Flowchart Detailing the Literature Review Process **eFigure 2.** Forest Plot of Odds of IV Thrombolysis **eFigure 3.** Forest Plot of Differences in Age **eFigure 4.** Forest Plot of Odds of Diabetes **eFigure 5.** Forest Plot of Odds of Hypertension **eFigure 6.** Forest Plot of Odds of Coronary Artery Disease **eFigure 7.** Forest Plot of Odds of Smoking **eFigure 8.** Forest Plot of Odds of Male Sex **eFigure 9.** Funnel Plot for 90-Day mRS 0-2 Analysis **eFigure 10.** Influence Diagnostics to Identify Outlier Studies in 90-Day mRS 0-2 Analysis **eFigure 11.** Influence Analysis to Identify Outlier Studies in TICI 2b-3 Analysis **eFigure 12.** Funnel Plot for Mortality Analysis **eAppendix.** Search Strategy **eTable.** Detailed Risk of Bias Analysis

Supplement 2.
Data Sharing Statement
