# Supplementary material for: Outcomes of Patients With Atrial Fibrillation Following Thrombectomy for Stroke: A Systematic Review and Meta-analysis
Source: JAMA Netw Open. 2023 Jan 6;6(1):e2249993. doi: 10.1001/jamanetworkopen.2022.49993 (PMC9857225; doi:10.1001/jamanetworkopen.2022.49993)
Supplement: Supplement 1. — eFigure 1. Flowchart Detailing the Literature Review Process eFigure 2. Forest Plot of Odds of IV Thrombolysis eFigure 3. Forest Plot of Differences in Age eFigure 4. Forest Plot of Odds of Diabetes eFigure 5. Forest Plot of Odds of Hypertension eFigure 6. Forest Plot of Odds of Coronary Artery Disease eFigure 7. Forest Plot of Odds of Smoking eFigure 8. Forest Plot of Odds of Male Sex eFigure 9. Funnel Plot for 90-Day mRS 0-2 Analysis eFigure 10. Influence Diagnostics to Identify Outlier Studies in 90-Day mRS 0-2 Analysis eFigure 11. Influence Analysis to Identify Outlier Studies in TICI 2b-3 Analysis eFigure 12. Funnel Plot for Mortality Analysis eAppendix. Search Strategy eTable. Detailed Risk of Bias Analysis [file jamanetwopen-e2249993-s001.pdf]

Supplemental Online Content

Kobeissi H, Ghozy S, Seymour T, et al. Outcomes of patients with atrial fibrillation following thrombectomy for stroke: a systematic review and meta-analysis. *JAMA Netw Open*. 2023;6(1):e2249993. doi:10.1001/jamanetworkopen.2022.49993

eFigure 1. Flowchart Detailing the Literature Review Process

eFigure 2. Forest Plot of Odds of IV Thrombolysis

eFigure 3. Forest Plot of Differences in Age

eFigure 4. Forest Plot of Odds of Diabetes

eFigure 5. Forest Plot of Odds of Hypertension

eFigure 6. Forest Plot of Odds of Coronary Artery Disease

eFigure 7. Forest Plot of Odds of Smoking

eFigure 8. Forest Plot of Odds of Male Sex

eFigure 9. Funnel Plot for 90-Day mRS 0-2 Analysis

eFigure 10. Influence Diagnostics to Identify Outlier Studies in 90-Day mRS 0-2 Analysis

eFigure 11. Influence Analysis to Identify Outlier Studies in TICI 2b-3 Analysis

eFigure 12. Funnel Plot for Mortality Analysis

eAppendix. Search Strategy

eTable. Detailed Risk of Bias Analysis

This supplemental material has been provided by the authors to give readers additional information about their work.

© 2023 Kobeissi H et al. *JAMA Network Open.*

eFigure 1. Flowchart Detailing the Literature Review Process

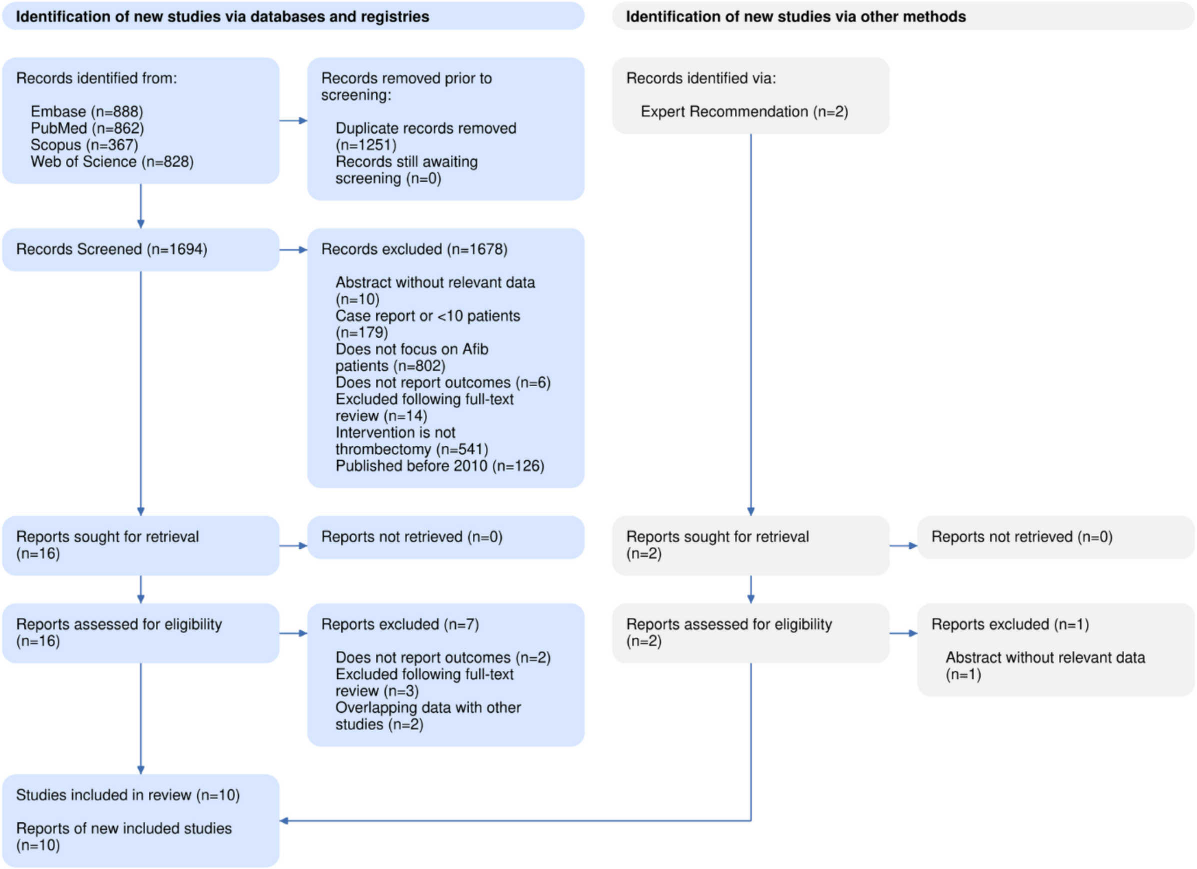

© 2023 Kobeissi H et al. *JAMA Network Open.*

eFigure 2. Forest Plot of Odds of IV Thrombolysis

Study	Afib Events	Afib Total	No Afib Events	No Afib Total	OR	95%−CI	Weight
Akbik et al., 2021	651	1517	1340	2652	0.74	[0.65; 0.84]	20.0%
Huang et al., 2021	33	123	50	122	0.53	[0.31; 0.90]	13.1%
Leker et al., 2020	24	109	32	121	0.79	[0.43; 1.44]	11.9%
Lin et al., 2020	24	43	15	40	2.11	[0.87; 5.07]	8.1%
Smaal et al., 2020	164	224	395	443	0.33	[0.22; 0.51]	15.2%
Fu et al., 2021	53	171	79	178	0.56	[0.36; 0.87]	14.9%
Churojana et al., 2018	10	50	19	84	0.86	[0.36; 2.02]	8.3%
Zdraljevic et al., 2022	11	62	21	65	0.45	[0.20; 1.04]	8.6%
**Random effects model**	**970**	**2299**	**1951**	**3705**	**0.64**	**[0.46; 0.88]**	**100.0%**

Heterogeneity: $I^2 = 68\%$, $\tau^2 = 0.130$, $p = 0.003$
Test for overall effect: $z = -2.738$ ($p = 0.006$)

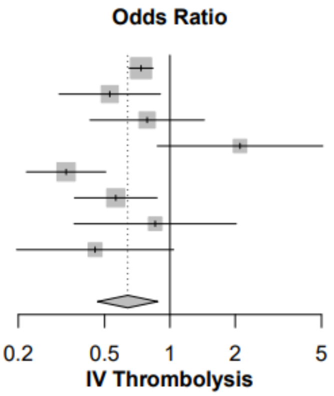

© 2023 Kobeissi H et al. *JAMA Network Open.*

eFigure 3. Forest Plot of Differences in Age

Study	Afib Total	Afib Mean	Afib SD	No Afib Total	No Afib Mean	No Afib SD	MD	95%−CI	Weight
Akbik et al., 2021	1517	76.00	11.0000	2652	65.00	15.0000	11.00	[10.20; 11.80]	18.7%
Lasek−Bal et al., 2022	108	74.92	9.2045	309	66.83	14.5238	8.08	[5.71; 10.46]	15.3%
Leker et al., 2020	109	75.05	11.7576	121	64.50	15.1000	10.55	[7.07; 14.03]	12.4%
Lin et al., 2020	43	72.60	9.5000	40	70.90	17.3000	1.70	[−4.37; 7.77]	7.2%
Smaal et al., 2020	224	72.80	10.1000	443	63.10	13.7000	9.70	[7.86; 11.54]	16.7%
Fu et al., 2021	171	77.00	9.7195	178	61.00	31.3909	16.00	[11.16; 20.84]	9.3%
Churojana et al., 2018	50	69.20	12.9000	84	60.20	16.0000	9.00	[4.05; 13.95]	9.1%
Zdraljevic et al., 2022	62	73.33	9.4873	65	59.83	13.2677	13.50	[9.50; 17.50]	11.2%
**Random effects model**	**2284**			**3892**			**10.17**	**[8.11; 12.23]**	**100.0%**

Heterogeneity: $I^2 = 68\%$, $\tau^2 = 5.741$, $p = 0.003$
Test for overall effect: $z = 9.678$ ($p < 0.001$)

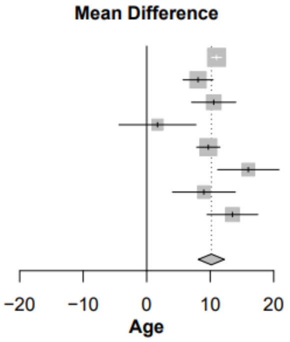

© 2023 Kobeissi H et al. *JAMA Network Open.*

eFigure 4. Forest Plot of Odds of Diabetes

Study	Afib Events	Afib Total	No Afib Events	No Afib Total	OR	95%−CI	Weight
Akbik et al., 2021	453	1517	721	2652	1.14	[0.99; 1.31]	76.9%
Leker et al., 2020	34	109	39	121	0.95	[0.55; 1.66]	4.8%
Lin et al., 2020	11	43	8	40	1.38	[0.49; 3.87]	1.4%
Smaal et al., 2020	47	224	67	443	1.49	[0.99; 2.25]	8.7%
Fu et al., 2021	44	171	44	178	1.06	[0.65; 1.71]	6.4%
Zdraljevic et al., 2022	12	62	10	65	1.32	[0.52; 3.32]	1.8%
**Random effects model**	**601**	**2126**	**889**	**3499**	**1.16**	**[1.02; 1.31]**	**100.0%**

Heterogeneity: $I^2 = 0\%$, $\tau^2 = 0$, $p = 0.810$
Test for overall effect: $z = 2.345$ ($p = 0.019$)

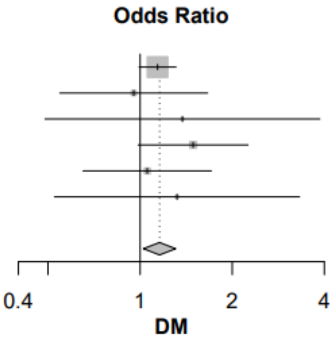

© 2023 Kobeissi H et al. *JAMA Network Open.*

eFigure 5. Forest Plot of Odds of Hypertension

Study	Afib Events	Afib Total	No Afib Events	No Afib Total	OR	95%−CI	Weight
Akbik et al., 2021	1252	1517	1860	2652	2.01	[1.72; 2.35]	48.4%
Leker et al., 2020	78	109	79	121	1.34	[0.76; 2.34]	9.7%
Lin et al., 2020	28	43	28	40	0.80	[0.32; 2.01]	3.9%
Smaal et al., 2020	154	224	215	443	2.33	[1.66; 3.27]	21.5%
Fu et al., 2021	130	171	120	178	1.53	[0.96; 2.45]	12.9%
Zdraljevic et al., 2022	55	62	47	65	3.01	[1.16; 7.83]	3.6%
**Random effects model**	**1697**	**2126**	**2349**	**3499**	**1.89**	**[1.57; 2.27]**	**100.0%**

Heterogeneity: $I^2 = 40\%$, $\tau^2 = 0.012$, $p = 0.138$

Test for overall effect: $z = 6.670$ ($p < 0.001$)

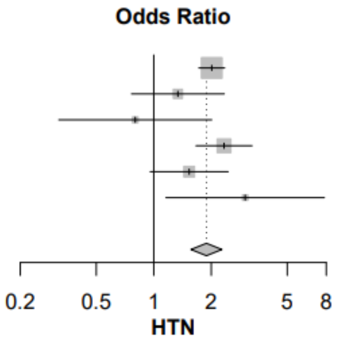

© 2023 Kobeissi H et al. *JAMA Network Open.*

eFigure 6. Forest Plot of Odds of Coronary Artery Disease

Study	Afib Events	Afib Total	No Afib Events	No Afib Total	OR	95%−CI	Weight
Lin et al., 2020	9	43	8	40	1.06	[0.36; 3.08]	51.0%
Churojana et al., 2018	4	50	21	84	0.26	[0.08; 0.81]	49.0%
**Random effects model**	**13**	**93**	**29**	**124**	**0.53**	**[0.14; 2.10]**	**100.0%**

Heterogeneity: $I^2 = 68\%$, $\tau^2 = 0.665$, $p = 0.078$
Test for overall effect: $z = -0.899$ ($p = 0.369$)

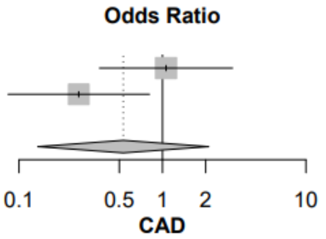

© 2023 Kobeissi H et al. *JAMA Network Open.*

eFigure 7. Forest Plot of Odds of Smoking

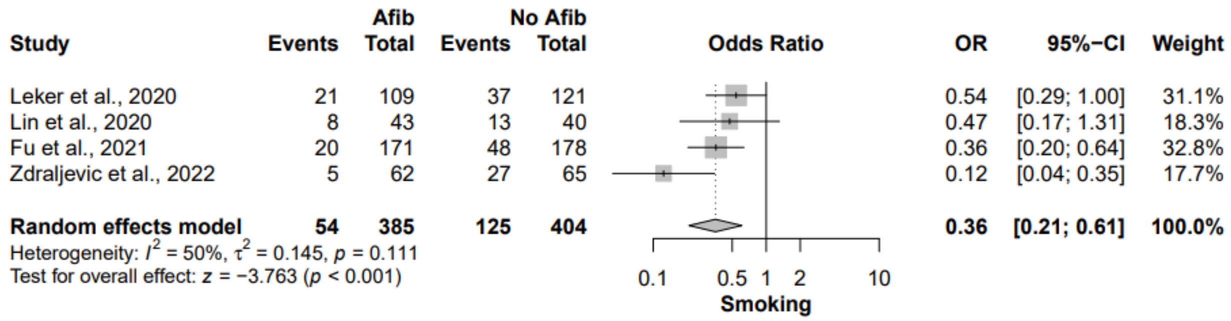

© 2023 Kobeissi H et al. *JAMA Network Open.*

eFigure 8. Forest Plot of Odds of Male Sex

Study	Afib Events	Afib Total	No Afib Events	No Afib Total	OR	95%−CI	Weight
Akbik et al., 2021	640	1517	1352	2651	0.70	[0.62; 0.80]	35.9%
Lasek−Bal et al., 2022	49	108	169	309	0.69	[0.44; 1.07]	11.5%
Leker et al., 2020	43	109	68	121	0.51	[0.30; 0.86]	8.7%
Lin et al., 2020	20	43	23	40	0.64	[0.27; 1.53]	3.7%
Smaal et al., 2020	117	224	226	443	1.05	[0.76; 1.45]	17.6%
Fu et al., 2021	83	171	104	178	0.67	[0.44; 1.02]	12.2%
Churojana et al., 2018	27	50	51	84	0.76	[0.37; 1.54]	5.3%
Zdraljevic et al., 2022	27	62	41	65	0.45	[0.22; 0.92]	5.2%
**Random effects model**	**1006**	**2284**	**2034**	**3891**	**0.71**	**[0.60; 0.84]**	**100.0%**

Heterogeneity: $I^2 = 22\%$, $\tau^2 = 0.018$, $p = 0.252$

Test for overall effect: $z = -3.868$ ($p < 0.001$)

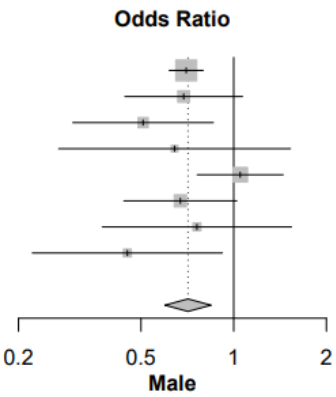

© 2023 Kobeissi H et al. *JAMA Network Open.*

eFigure 9. Funnel Plot for 90-Day mRS 0-2 Analysis

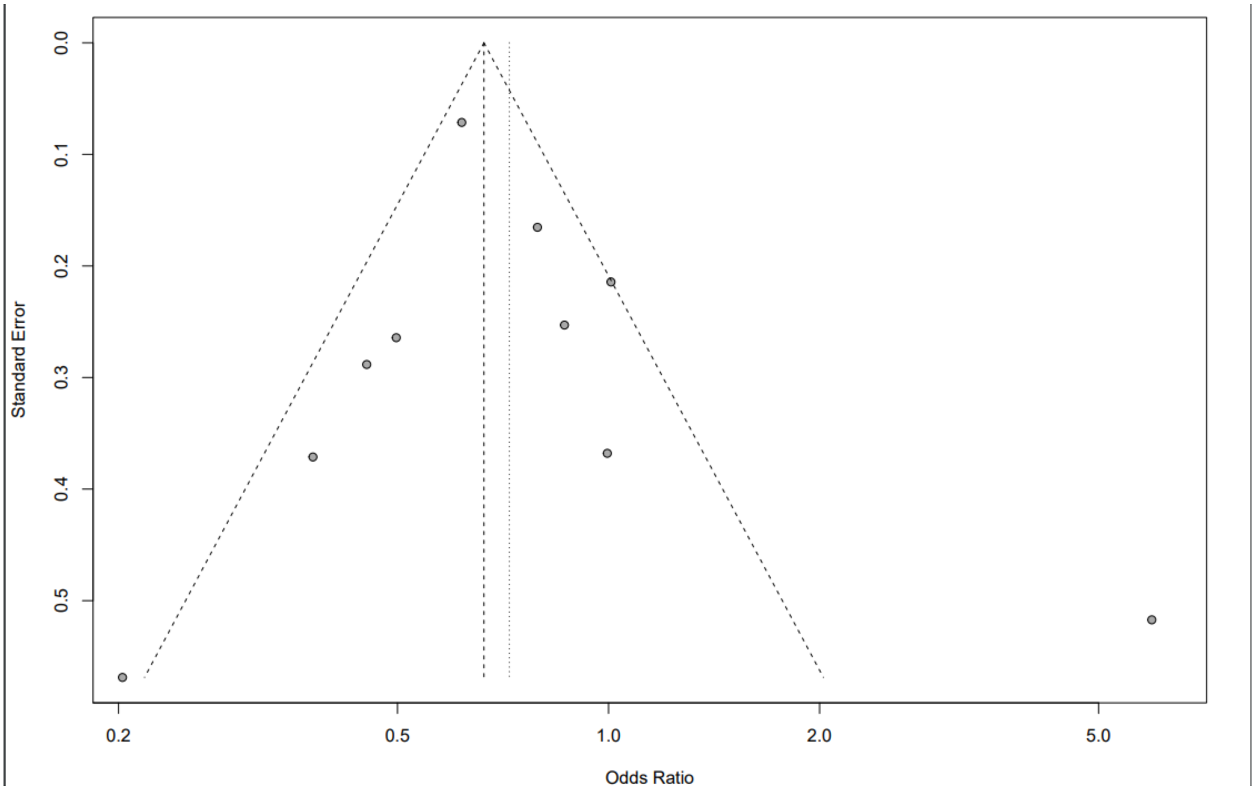

© 2023 Kobeissi H et al. *JAMA Network Open.*

eFigure 10. Influence Diagnostics to Identify Outlier Studies in 90-Day mRS 0-2 Analysis

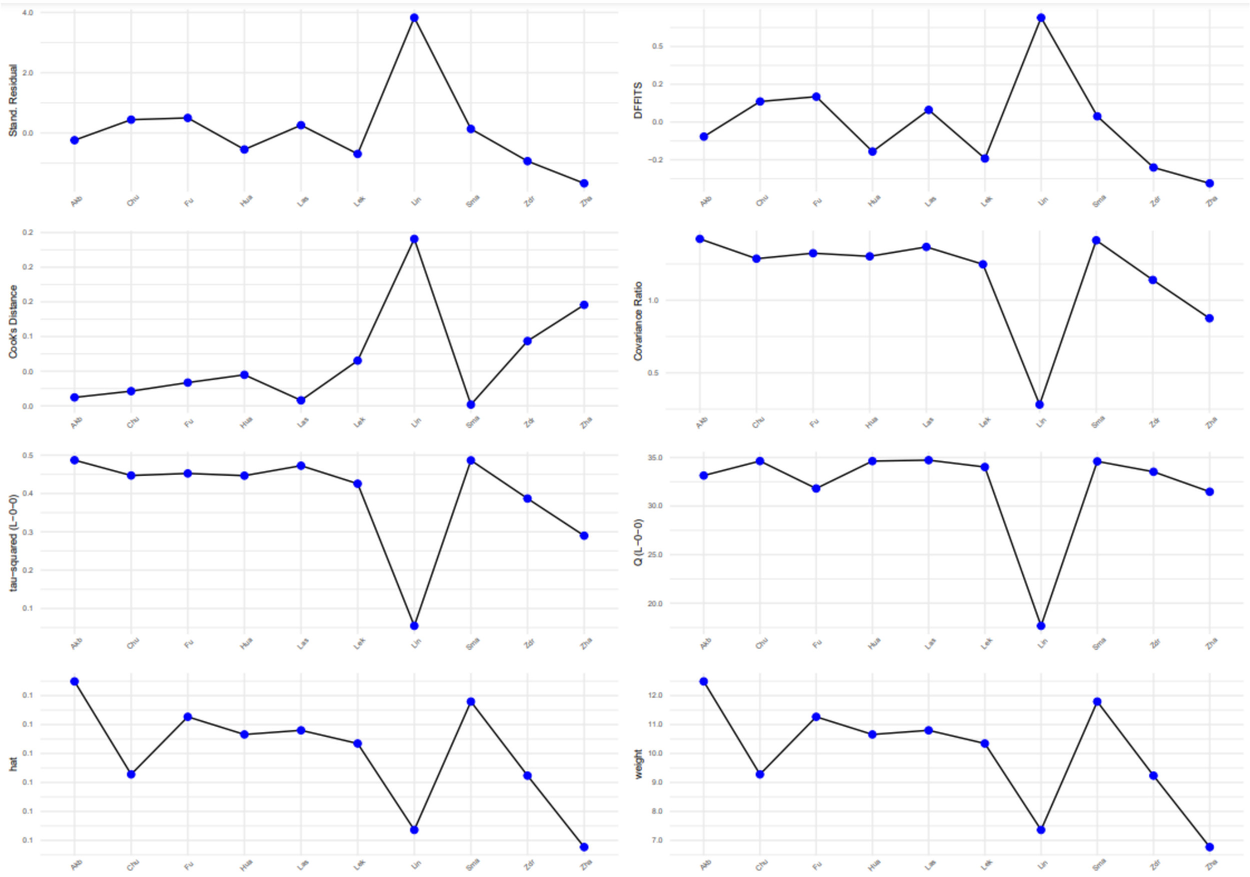

© 2023 Kobeissi H et al. *JAMA Network Open.*

eFigure 11. Influence Analysis to Identify Outlier Studies in TICI 2b-3 Analysis

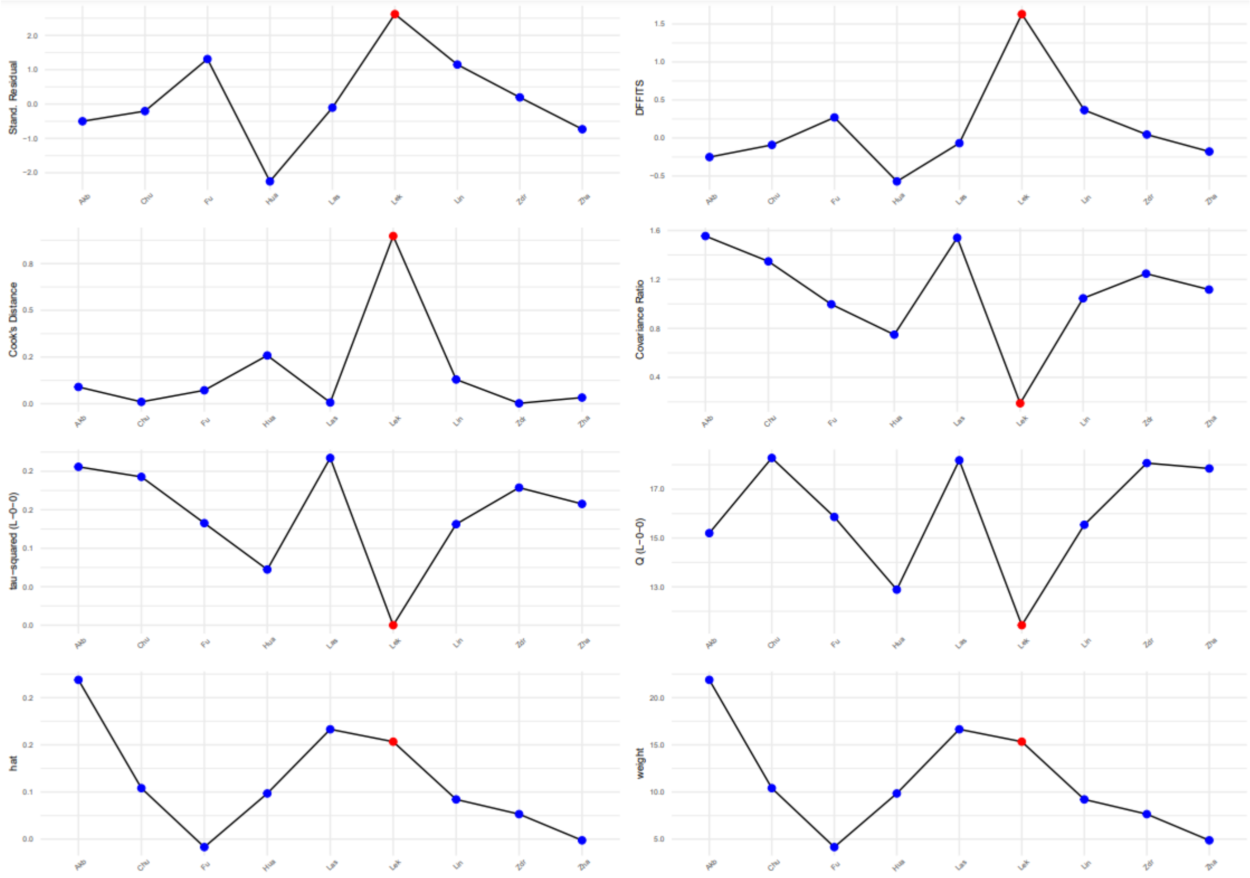

© 2023 Kobeissi H et al. *JAMA Network Open.*

eFigure 12. Funnel Plot for Mortality Analysis

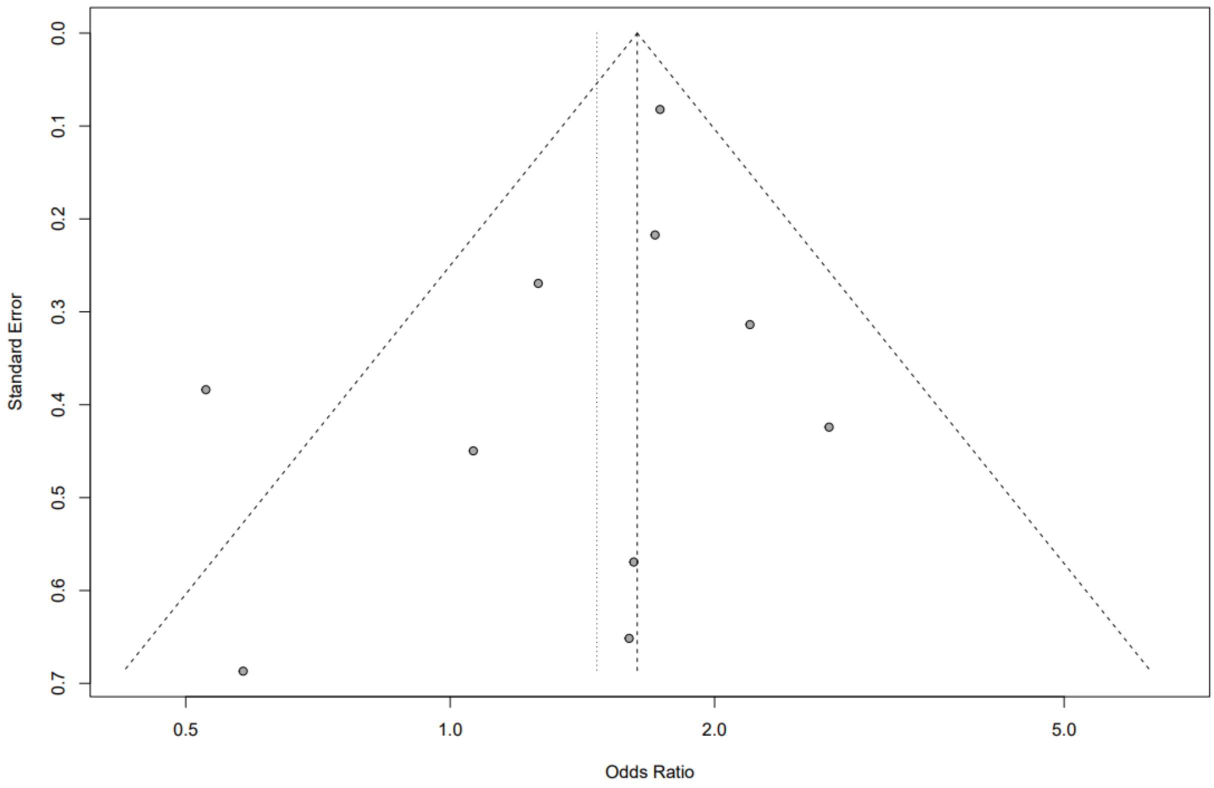

© 2023 Kobeissi H et al. *JAMA Network Open.*

eAppendix. Search Strategy

Web of Science: ((TS=((((("stroke" OR "stroke") OR "strokes") OR "stroke s") OR (((("cerebral infarction" OR ("cerebral" AND "infarction")) OR "cerebral infarction") OR ("cerebral" AND "infarct")) OR "cerebral infarct")) OR ((((((((("infarctation" OR "infarcted") OR "infarctic") OR "infarcting") OR "infarction") OR "infarction") OR "infarct") OR "infarctions") OR "infarcts") OR "infarctive"))) AND TS=(((("thrombectomy" OR "thrombectomy") OR "thrombectomies") OR (((((("mechanical" OR "mechanically") OR "mechanicals") OR "mechanics") OR "mechanics") OR "mechanic") AND (("thrombectomy" OR "thrombectomy") OR "thrombectomies"))) OR ("endovascular" AND (((((("therapeutics" OR "therapeutics") OR "therapies") OR "therapy") OR "therapy") OR "therapy s") OR "therapys")))) AND TS=(("Atrial Fibrillation" OR "AF" OR "AFib"))

Embase: Embase <1974 to 2022 July 13> 1 ("stroke" or "stroke" or "strokes" or "stroke s" or ("cerebral infarction" or ("cerebral" and "infarction") or "cerebral infarction" or ("cerebral" and "infarct") or "cerebral infarct") or ("infarctation" or "infarcted" or "infarctic" or "infarcting" or "infarction" or "infarction" or "infarct" or "infarctions" or "infarcts" or "infarctive")).ab,ti. 798870 2 ("thrombectomy" or "thrombectomy" or "thrombectomies" or (("mechanical" or "mechanically" or "mechanicals" or "mechanics" or "mechanics" or "mechanic") and ("thrombectomy" or "thrombectomy" or "thrombectomies")) or ("endovascular" and ("therapeutics" or "therapeutics" or "therapies" or "therapy" or "therapy" or "therapy s" or "therapys"))).ab,ti. 41213 3 ("Atrial Fibrillation" or "AF" or "AFib").ab,ti. 175490 4 1 and 2 and 3 911

Scopus: TITLE-ABS ((((("stroke" OR "stroke") OR "strokes") OR "stroke s") OR (((("cerebral infarction" OR ("cerebral" AND "infarction")) OR "cerebral infarction") OR ("cerebral" AND "infarct")) OR "cerebral infarct")) OR ((((((((("infarctation" OR "infarcted") OR "infarctic") OR "infarcting") OR "infarction") OR "infarction") OR "infarct") OR "infarctions") OR "infarcts") OR "infarctive") AND ((("thrombectomy" OR "thrombectomy") OR "thrombectomies") OR (((((("mechanical" OR "mechanically") OR "mechanicals") OR "mechanics") OR "mechanics") OR "mechanic") AND (("thrombectomy" OR "thrombectomy") OR "thrombectomies"))) OR ("endovascular" AND (((((("therapeutics" OR "therapeutics") OR "therapies") OR "therapy") OR "therapy") OR "therapy s") OR "therapys")) AND ("Atrial Fibrillation" OR "AF" OR "AFib"))

PubMed: (((("stroke"[MeSH Terms] OR "stroke"[All Fields]) OR "strokes"[All Fields]) OR "stroke s"[All Fields]) OR (((("cerebral infarction"[MeSH Terms] OR ("cerebral"[All Fields] AND "infarction"[All Fields])) OR "cerebral infarction"[All Fields]) OR ("cerebral"[All Fields] AND "infarct"[All Fields])) OR "cerebral infarct"[All Fields])) OR ((((((((("infarctation"[All Fields] OR "infarcted"[All Fields]) OR "infarctic"[All Fields]) OR "infarcting"[All Fields]) OR "infarction"[MeSH Terms]) OR "infarction"[All Fields]) OR "infarct"[All Fields]) OR "infarctions"[All Fields]) OR "infarcts"[All Fields]) OR "infarctive"[All Fields]) AND ((("thrombectomy"[MeSH Terms] OR "thrombectomy"[All Fields]) OR "thrombectomies"[All Fields]) OR (((((("mechanical"[All Fields] OR "mechanically"[All Fields]) OR "mechanicals"[All Fields]) OR "mechanics"[MeSH Terms]) OR "mechanics"[All Fields]) OR

© 2023 Kobeissi H et al. *JAMA Network Open.*

"mechanic"[All Fields]) AND (("thrombectomy"[MeSH Terms] OR "thrombectomy"[All Fields]) OR "thrombectomies"[All Fields]))) OR ("endovascular"[All Fields] AND (((((("therapeutics"[MeSH Terms] OR "therapeutics"[All Fields]) OR "therapies"[All Fields]) OR "therapy"[MeSH Subheading]) OR "therapy"[All Fields]) OR "therapy s"[All Fields]) OR "therapys"[All Fields])) AND ("Atrial Fibrillation" OR "AF" OR "AFib")

eTable. Detailed Risk of Bias Analysis

		Year	Sample (n)	New Castle Ottawa scale assessment (NOS)								
				Selection				Comparability	Outcome			
	Study	Year	Sample (n)	Representativeness of the exposed cohort	Selection of the non exposed cohort	Ascertainment of exposure	Demonstration that outcome of interest was not present at start of study	Comparability of cohorts on the basis of the design or analysis	Assessment of outcome	Was follow-up long enough for outcomes to occur	Adequacy of follow up of cohorts	Quality Score
1	Akbik	2021	4,169	*	*	*		*	*	*	*	Good
2	Ždraljević	2022	127	*	*	*		*	*	*	*	Good
3	Huang	2021	245	*	*	*		*	*	*	*	Good
4	Lasek-Bal	2022	417	*	*	*		*	*	*	*	Good
5	Leker	2020	230	*	*	*		*	*	*	*	Good
6	Lin	2020	83		*	*		*	*	*	*	Fair
7	Smaal	2020	666	*	*	*		*	*	*	*	Good
8	Fu	2021	394	*	*	*		*	*	*	*	Good
9	Churojana	2018	134	*	*	*		*	*	*	*	Good
10	Zhang	2019	78		*	*		*	*	*	*	Fair

© 2023 Kobeissi H et al. *JAMA Network Open.*
