# Supplementary material for: Outcomes of Patients With Atrial Fibrillation Following Thrombectomy for Stroke: A Systematic Review and Meta-analysis
Source: JAMA Netw Open. 2023 Jan 6;6(1):e2249993. doi: 10.1001/jamanetworkopen.2022.49993 (PMC9857225; doi:10.1001/jamanetworkopen.2022.49993)
Supplement: Supplement 2. — Data Sharing Statement [file jamanetwopen-e2249993-s002.pdf]

Data Sharing Statement

Kobeissi. Outcomes of Patients With Atrial Fibrillation Following Thrombectomy for Stroke. *JAMA Netw Open*. Published January 06, 2023. doi:10.1001/jamanetworkopen.2022.49993

Data
Data available: No

Additional Information
Explanation for why data not available: The data that support this study comes from publicly available papers, which are cited in our systematic review and meta-analysis.
